# 基于三维重建的影像学分析在部分实性肺结节中的研究进展

**DOI:** 10.3779/j.issn.1009-3419.2022.101.03

**Published:** 2022-02-20

**Authors:** 子成 刘, 赫 杨, 鸿亚 王, 亮 陈, 全 朱

**Affiliations:** 210029 南京，南京医科大学第一附属医院胸外科 Department of Thoracic Surgery, The First Affiliated Hospital of Nanjing Medical University, Nanjing 210029, China

**Keywords:** 肺肿瘤, 三维重建, 实性成分, 预后, Lung neoplasms, Three-dimensional reconstruction, Solid component, Prognosis

## Abstract

中国肺癌的发病率和死亡率居所有恶性肿瘤之首，随着高分辨率计算机断层扫描（computed tomography, CT）在临床的普及，胸部CT已成为临床筛查早期肺癌、降低肺癌死亡率的重要手段。早期肺腺癌的影像学常表现为含有磨玻璃成分的部分实性结节。随着影像学的发展，部分实性结节的影像学表现与其预后的关系越来越受关注。同时随着三维重建技术的发展，提高了临床医师对该类结节诊断治疗的精确性。本文聚焦于部分实性结节的传统影像学分析和基于三维重建的影像学分析，对两者的利弊作一系统阐述。

## Abstract (en)

The incidence and mortality of lung cancer rank first among all malignant tumors in China. With the popularization of high resolution computed tomography (CT) in clinic, chest CT has become an important means of clinical screening for early lung cancer and reducing the mortality of lung cancer. Imaging findings of early lung adenocarcinoma often show partial solid nodules with ground glass components. With the development of imaging, the relationship between the imaging features of some solid nodules and their prognosis has attracted more and more attention. At the same time, with the development of 3D-reconstruction technology, clinicians can improve the accuracy of diagnosis and treatment of such nodules.This article focuses on the traditional imaging analysis of partial solid nodules and the imaging analysis based on 3D reconstruction, and systematically expounds the advantages and disadvantages of both.

随着对非小细胞肺癌（non-small cell lung cancer, NSCLC）研究的不断加深以及影像技术、人工智能的快速发展，越来越多的胸外科医生认识到影像学特征在NSCLC的诊断中发挥着不可替代的作用，具有相当大的预后价值。日本临床肿瘤学组（Japan Clinical Oncology Group, JCOG）的一系列前瞻性研究首先验证了影像学特征对病理侵袭性的预测作用^[1]^。近年来，低剂量计算机断层扫描（low dose computed tomography, LDCT）的普及改变了中国肺癌筛查的主要方式，在中年人群体中，LDCT逐渐替代胸部X射线成为肺癌筛查的首选方式。同时，作为新型冠状病毒筛查的手段之一，其他疾病为主诉的住院患者筛查出肺结节或肺部肿物也不占少数。磨玻璃阴影（ground glass opacity, GGO）、支气管空气征、胸膜牵拉征等传统CT特征对NSCLC的病理侵袭程度与基因突变状态的预测作用已被大量研究证实^[2, 3]^；影像组学作为近年来的研究热点，也有不少研究建立了NSCLC相关的预测模型^[4, 5]^。在本篇综述中，我们收集了近年来关于以GGO为主要特征的NSCLC的相关文献，重点关注了传统二维CT和基于三维重建的影像学分析分别对早期NSCLC的诊断与预后的预测价值，以总结概述其中的关系以及优劣。

随着肿瘤组织逐渐浸润及侵犯程度的加深，会出现肿瘤间质内纤维化或炎症反应等病理特征，从而在影像上表现一些特征。磨玻璃结节分为两类：纯磨玻璃结节（pure ground glass nodule, pGGN）和混杂性磨玻璃结节（mixed ground glass nodule, mGGN）。其中前者磨玻璃影边界清楚，呈圆形或类圆形，平均CT值一般较低，病理上通常表现为不典型腺瘤样增生或原位腺癌为主，预后优秀。而mGGN则是一个复杂的种类，实性成分的增多往往与肿瘤的发展相关，同时形态上也具有很多特征，病理的种类也是各不相同，因此是一类需要重点研究的肺癌。早期对于NSCLC的研究主要是通过胸外科医生来肉眼评估，来评价结节的特征，如肿瘤大小、边缘是否光滑、分叶征，毛刺征、支气管空气征、血管集束征、胸膜牵拉征等（图 1）。通过对这些特征也可以一定程度上反应早期NSCLC的病理侵袭程度。

**图 1:**
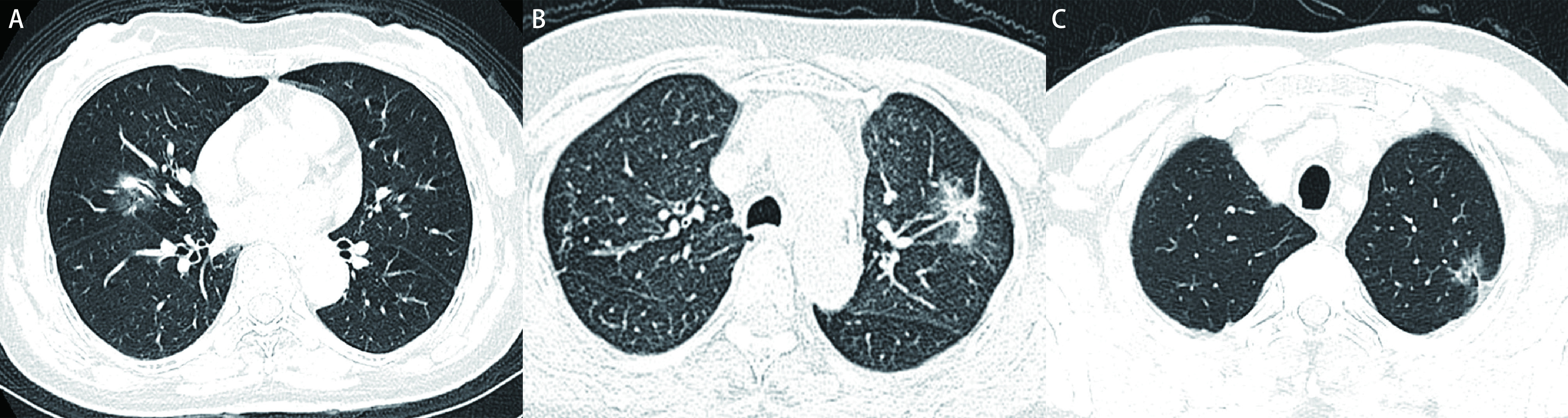
常见的肺癌相关影像学特征。A：支气管充气征；B：血管集束征；C：胸膜牵拉征 Common radiographic features associated with lung cancer. A: Air bronchogram; B: Vessel convergence sign; C: Pleural indentation.

## 1 传统影像学对于部分实性结节的研究

早期NSCLC在CT上通常表现为具有GGO成分的小结节。近年来大量研究提出GGO成分的有无被认为是判断肿瘤预后的一个重要指标。Hattori等^[6-8]^通过对JCOG0201数据集的分析，认为GGO成分是NSCLC良好预后的因素之一。该研究提出将具有GGO成分的NSCLC与无GGO成分的实体NSCLC应分为两种肿瘤类别，并且GGO成分的有无应作为下一版临床T分期的重要参数。相同的结论也被证实在对于T1期NSCLC与多灶原发性NSCLC的研究中。同时，也有很多文章提出实体成分的大小对NSCLC的预后作用，但对于实体部分的测量目前并没有一个统一的共识。第一，测量实型成分的窗宽窗位。根据国际肺癌研究协会（International Association for the Study of Lung Cancer, IASLC）和2017年Fleischner学会放射学肺结节测量指南，临床T分期中，应通过CT上的肺窗来测量亚实性肺结节的实体成分，本质上是用较宽的窗宽来显示肺实质以及其异常的病理变化^[9, 10]^。但是也有学者提出在肺窗上对实体成分的测量一致性较低，而纵隔窗上由于与背景有明显区分，便不会出现这种问题^[11]^。总的来说，尽管通过纵隔窗的测量方法，一定程度上会改变部分患者的临床T分期，但是对于预后的评价作用，就目前的研究来说没有发现额外的收益^[12, 13]^。除了临床分期，传统影像学对淋巴结转移状态也有一定的帮助，Aokage等^[14]^利用JCOG0201的数据库，研究了可能与淋巴结阳性相关的影像学特征，利用肿瘤直径、实性肿瘤比（consolidation tumor ratio, CTR）和GGO成分的有无构建出预测模型，灵敏性和特异性分别可达到95.4%和40.5%，并通过外部队列验证了其有效性。Fu等^[15]^的研究中，主要探究了不同肿瘤大小与GGO有无对淋巴结状态的影响，发现随着肿瘤直径的变大，淋巴结转移的比例随之升高，而在纯GGO且小于1 cm的肺结节中，出现淋巴结转移的例数为0。当然由于该研究样本量较少，对于纯GGO且小于1 cm的肺结节，是否可以考虑不行淋巴结的清扫或采样还有待更大样本量的高质量研究来证实。通过对CT上各种放射性特征的识别和随访CT的变化，往往就可以反映患者的预后。Hattori等^[16]^纳入了1, 181例可切除的NSCLC患者，去分析CTR，肿瘤大小以及固体成分大小与患者5年生存期的关系，对于混合磨玻璃结节的患者，结节最大径与CTR均与较差的总生存率无关（*P*=0.637, 0; *P*=0.139, 5）。但是新版的肿瘤原发灶-淋巴结-转移（tumor-node-metastasis, TNM）分期中，肿瘤大小和实性成分共同决定了患者的T分期，并且对患者的治疗与预后有着决定性的影响。有研究表明，出现复发的肺癌患者在二维实体成分大小、三维实体成分最大直径和三维实体成分明显高于无复发的患者（*P* < 0.001, *P* < 0.001, *P*=0.023）^[17]^。在病理侵袭性方面，也有多项研究^[18, 19]^发现肺腺癌中肿瘤的实体大小或实性成分比例与肿瘤的侵袭性以及预后相关。对于可切除的NSCLC，目前有许多病理或临床特征已被报道可以影响患者的预后，而对于亚实性结节，薄层CT上的实体成分或实体成分所占比往往是至关重要的。Alpert等^[20]^将浸润性肺腺癌根据病理亚型分为贴壁为主型组和侵袭性更强的其余浸润性腺癌组，进行结节分析，发现两组间在最大直径，实性成分体积以及实性成分百分比上存在明显差异，并通过多因素回归模型计算出实体成分每增加1%，结节出现非贴壁为主的浸润性肺腺癌的概率要增加约17%，特异性高达97.7%。此外，在早期NSCLC的随访中，实体成分的倍增时间对患者的预后也被证实有较大的影响。Setojima等^[21]^在对可切除NSCLC的预后多因素回归分析研究中，发现实体成分倍增时间是患者的无复发生存时间的独立显著因素。因此，实体成分和实体成分倍增时间对于精准规划患者治疗方式和预测总体预后意义很大，并可以为可切除NSCLC患者选择合适的手术方式。

## 2 三维重建技术在传统影像学分析上的突破

相比于传统的X线、CT、磁共振成像（magnetic resonance imaging, MRI）等诊断方式，三维重建技术在许多方面都展现出了巨大的优势。通过对二维图像的三维化，使得医疗诊断不再完全依赖于医生的诊断经验去估计病灶的大小和形状。目前临床上比较常用的有Deepinsight、Horos、Mimics、3D synapse等三维重建软件，但是都各有利弊。在计算机上模拟出结节与周围组织结构的形态，并通过与软件处理工具相配合，使医生通过旋转、平移、结节分析等操作对病灶的性质和三维结构关系获得准确地了解，制定合理的手术方案（图 2）。

**图 2:**
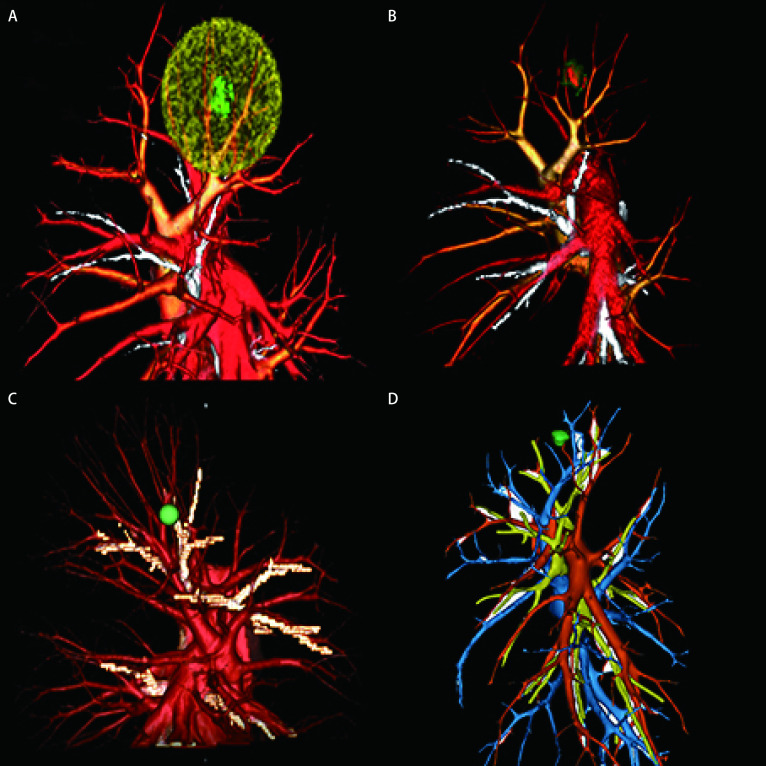
三维重建技术。A：切缘球制定手术规划；B：部分实性结节三维特征，红色代表实性成分，绿色代表磨玻璃成分；C：Horos对CT成像质量要求低，无法直接区分动静脉；D：Mimics操作时间长，对解剖知识储备要求高，动静脉区分度好。 3D reconstruction technology. A: Cutting edge balls for surgical planning; B: Part solid nodules three-dimensional reconstruction, red part represents solid components and green represents ground glass components; C: Horos has low requirements on CT imaging quality and cannot distinguish arteriovenous directly; D: Mimics needs long operation time, high requirement for anatomical knowledge reserve and good differentiation of arteriovenous. CT: computed tomography

### 2.1 肺结节三维体积测量以及实性成分测量

实性成分对于NSCLC的预后作用已经成为共识，然而目前，对于实体成分的表示方法尚且没有一个共识。目前比较常见的有实性部分最大直径、CTR、三维实性成分比例等。在JCOG的一系列前瞻性研究中，便是以CTR作为评价指标^[1, 22, 23]^，因此后续很多对于GGO的研究中，也都使用了CTR来作为测量指标。Fu等^[15]^回顾性分析了440例NSCLC病例，认为CTR可以用于预测病情与总生存期，且纯GGO患者都有较好的5年总是生存期。同时，CTR在Tsurugai等^[24]^的研究中也被发现可有效预测NSCLC患者立体定向放疗术后的无病生存期且CTR较低往往有较好的预后。但是由于受限于软件算法等原因，多采用二维影像上通过肉眼去测量，这就导致了可能存在的误差，且不同研究不同医师之间对亚实性GGO中实体成分的判别的一致性相差较大。更重要的是，早期对实体成分的测量是二维横截面的，而NSCLC往往在CT上并不是一个规则的结构，很多会出现分叶，毛刺等不规则的特征，这就导致在横截面上的最大直径往往并非肿瘤实际的最大直径，那其测量的实性成分往往与实际肿瘤的实性成分含量不符。当然随着计算机技术的发展，开发了很多三维重建软件，如DeepInsight重建系统，3D synapse重建软件（富士公司，日本东京）等，针对临床需要开发了针对肺结节的测量功能，可以在三维上重建出结节的同时，直接计算出GGO的实性成分与磨玻璃成分大小与比值，其真实性与准确性较传统的二维算法要高出许多。Kamiya等^[17]^对96例CT表现为亚实性结节的NSCLC患者进行比较，统计了包括二维最大直径，三维最大直径，结节体积与实性部分体积等指标，并于患者无病生存期进行回归分析，发现相比于其他影像学指标，实性部分体积是患者预后的独立预测指标。Li等^[25]^也收集了128例可切除腺癌患者的影像学资料，对比二维和三维的测量指标后，发现非浸润组的三维肿瘤总体积和实性成分体积均显著大于浸润性腺癌组，且高实性体积与肺腺癌浸润性显著相关。Furumoto等^[26]^在对Ia期肺NSCLC预后的影响因素研究中，多因素回归分析表明实体部分体积是唯一的预测因素而实体部分直径却不是，并且在比较实体部分体积与实体部分直径的受试者工作特征曲线下面积时，实体部分体积也具有一定的优势。这些研究充分肯定了基于三维重建的结节测量方式在对部分实性结节的诊断与治疗中的意义。相似的结果在Li等^[18]^的回顾性研究中得到证实，他们发现在T1N0M0的早期肺腺癌中，实性部分体积比值与贴壁为主的病理亚型百分比呈明显负相关，而贴壁型往往预示着较好的预后，因此更高的实性部分体积比往往预示着更高的病理侵袭性。

### 2.2 对亚实性结节影像学特征的三维评估

在三维重建系统将亚实性结节在三维上重建出来后，我们可以通过在软件中旋转、移动、切割后，从不同角度去观察结节本身的形态和特征，比如对于结节的形状是否规则、分叶、胸膜牵拉等。柯君等^[27]^对结节影响特征的二维表现和三维表现的比较研究中发现，三维重建后，在识别患者的支气管空气征、血管集束征、分叶征和胸膜牵拉等阳性率均高于二维CT平扫。这个原因可能是由于癌肿的生长方向往往是三维的，而且肺动脉在胸部CT上走行角度与静脉不同，多半为斜行的，在肺窗上往往表现为连续层面的高密度圆形血管影，在重建后，可以很好的体现非水平角度的生长方向和血管支气管穿行情况，因此可以发现一部分在二维CT平扫上被疏漏的部分。

### 2.3 定位结节位置与深度

除了对于结节特征的定量分析，三维重建的发展使得胸外科医师可以更好的定位结节的位置与深度。李响等^[28]^基于三维重建提出三维位置定义法，通过结节所在肺叶的叶支气管开口中心为O点，取结节中心为A点，连接OA并向外延长，与脏层胸膜相交于B点三点位置，提出深度比这一概念（图 3），通过深度比0%-33.3%、33.4%-66.6%、66.7%-100%来将结节分为外区、中区、内区，相较传统的二维位置定位法更加精确，更加有利于合理地进行术前规划和手术方式的确定。不仅如此，日本富士的3D synapse在三维重建的基础上研发出支气管镜模拟功能，该功能在重建出肺部支气管的同时，可以计算出与结节相距垂直距离最近支气管开口之间的距离，从而方便临床医师在支气管镜操作时选取最合适的路径。总而言之，三维重建可以使临床医师更加轻易而精准地识别结节，定位结节，并且制定最合适的治疗方案。

**图 3:**
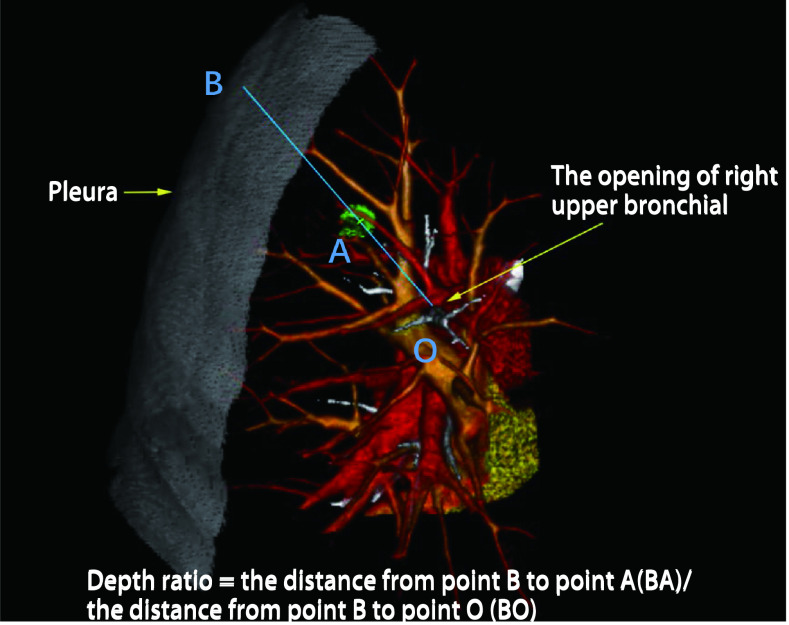
基于三维重建的肺结节三维位置定义法和深度比的定义 Definition of 3D position and depth ratio of pulmonary nodules based on 3D reconstruction

## 3 展望

我们认为尽管近几年影像组学飞速发展，可以从医学影像中提取大量肉眼难以发觉的数据，但是目前依旧尚处于开发阶段，对于不同数据提取工具和结果处理方式也都不同，尚未出现全面、公认有效的影像组学数据库及处理方法。与此同时，传统影像学分析依然是广大临床医师对NSCLC患者进行诊断和治疗的最根本方式。而现在，随着三维重建的发展，如同给传统影像学研究配备先进的武器，使得临床医师能够对病灶有更全面的认识，能够更好的实现精准医疗的概念。当然目前三维重建也依旧有许多可以改进的地方，如操作方式的简化、对低质量的影像资源的处理、人工智能制定手术方案等，笔者相信随着技术的不断发展，这些都能一一改善。最后，我们认为，目前基于三维重建的影像学研究还比较少，还有许多值得去挖掘的信息，这有待将来更多高质量的研究去完善这方面的空缺。
